# Comparison of Capture Rates of the National Cancer Database Across Race and Ethnicity

**DOI:** 10.1001/jamanetworkopen.2023.50237

**Published:** 2023-12-27

**Authors:** Yasoda Satpathy, Percival Nam, Matthew Moldovan, James D. Murphy, Luke Wang, Ithaar Derweesh, Brent S. Rose, Juan Javier-DesLoges

**Affiliations:** 1 Department of Urology, University of California San Diego School of Medicine, La Jolla; 2 Department of Radiation Medicine and Applied Science, University of California San Diego School of Medicine, La Jolla

## Abstract

**Importance:**

The National Cancer Database (NCDB) is an invaluable and widely used resource for cancer research, but the current state of representation of different racial and ethnic groups compared with the United States Cancer Statistics (USCS) database is unknown.

**Objective:**

To examine whether Hispanic and American Indian or Alaska Native individuals have lower representation in the NCDB compared with the USCS database.

**Design, Setting, and Participants:**

This multicenter, retrospective cohort study assessed individuals diagnosed with breast, colorectal, lung, and prostate cancer from January 1, 2004, to December 31, 2006, and January 1, 2017, to December 31, 2019, in the NCDB and USCS databases. Data analysis was performed from September 2022 to October 2023.

**Exposure:**

Time.

**Main Outcomes and Measures:**

The primary outcome was the absolute percentage change (APC) in capture rate across the study period.

**Results:**

The cohort included 5 175 007 individuals (0.50% American Indian or Alaska Native, 3.10% Asian or Pacific Islander, 12.01% Black, 6.58% Hispanic, and 77.81% White) who were diagnosed with breast, colorectal, lung, and prostate cancer. Capture rates were the lowest for individuals who were Hispanic (40.83% in 2004-2006 and 54.75% in 2017-2019; *P* < .001) or American Indian or Alaska Native (20.72% in 2004-2006 and 41.41% in 2017-2019; *P* < .001). The APCs were positive for both racial categories across all 4 cancers. However, overall APCs for Hispanic individuals (13.92%) remained lower than the overall APCs of White individuals (22.23%; *P* < .001). The APCs were greater for American Indian or Alaska Native individuals than for White individuals for prostate (14.68% vs 11.57%) and breast (21.61% vs 17.90%) cancer (*P* < .001), but the APCs for American Indian or Alaska Native individuals were lower than for White individuals for lung cancer (24.54% vs 33.03%; *P* < .001).

**Conclusions and Relevance:**

In this cohort study of individuals diagnosed with cancer in the NCDB, Hispanic and American Indian or Alaska Native individuals diagnosed with breast, colorectal, lung, and prostate cancer were undercaptured in the NCDB, but their representation improved over time. Increased study is needed to determine where these populations predominantly seek cancer care.

## Key Points

**Question:**

How well does the National Cancer Database represent Hispanic and American Indian or Alaska Native individuals who are diagnosed with breast, colorectal, lung, and prostate cancer?

**Findings:**

In this cohort study of 5 175 007 individuals diagnosed with breast, colorectal, lung, and prostate cancer, capture rates of historically underrepresented populations increased from 2004-2006 to 2017-2019 for Hispanic individuals (absolute percentage change, 13.92%) and American Indian or Alaska Native individuals (absolute percentage change, 20.69%).

**Meaning:**

The representation of Hispanic and American Indian or Alaska Native individuals in the National Cancer Database has increased over time.

## Teaser

This cohort study examined changes in the capture rate of the National Cancer Database of Hispanic individuals and American Indian or Alaska Native individuals diagnosed with breast, colorectal, lung, and prostate cancer.

## Introduction

Through the efforts of the American Cancer Society (ACS) and the American College of Surgeons’ Commission on Cancer (CoC), the creation and maintenance of the National Cancer Database (NCDB) has led to the development of innumerable studies on cancer.^1,2^ The NCDB is a database of all individuals who have been diagnosed with cancer at more than 1500 CoC-accredited facilities.^3^ The database has expanded rapidly since its inception in 1989, from capturing only 57% of the estimated number of US cancer cases in 1994 to capturing 72% of all US cancer cases in 2021.^3,4^ The NCDB is an invaluable resource, and many of these studies are being used in clinical guideline development.

Given the widespread use of this clinical cancer registry, it is imperative to monitor the representation of different racial and ethnic groups that are treated at CoC-accredited hospitals. Because NCDB data are derived only from CoC-accredited facilities, these cases are influenced by numerous geographic and sociodemographic factors.^2,5^ Furthermore, Black individuals and individuals from low socioeconomic backgrounds use CoC-accredited facilities less regularly, which indicates that the NCDB may not be generalizable to certain cohorts.^6^

Capture rates are the ratio of the number of NCDB cases to the number of United States Cancer Statistics (USCS) cases for a given population.^7^ The NCDB capture rates for various racial groups are well characterized for genitourinary cancers, revealing that American Indian or Alaska Native and Asian or Pacific Islander individuals have the lowest capture rates.^7^ However, it is unknown how capture rates have changed for nongenitourinary cancers among American Indian or Alaska Native individuals or for individuals who are Hispanic. Previous work^8^ found that the capture rate for Hispanic individuals is 51.1%. Numerous factors may contribute to this lack of representation, including structural racism; barriers to care, such as low socioeconomic status and language differences; and greater probability of living in underserved or rural communities with less access to CoC-accredited facilities.^9,10,11,12^ The contribution of such factors combined with decreased cancer screening and health insurance coverage rates leads to adverse health outcomes in Hispanic populations.^13,14,15^ Therefore, it is important to clarify whether databases such as the NCDB sufficiently represent American Indian or Alaska Native and Hispanic populations.

The aim of this study was to examine the capture rate of the NCDB by comparing it to the USCS database. The last analysis of capture rates of the NCDB compared with the USCS database was performed by Lerro et al^8^ using the 2004-2006 NCDB Participant User File; thus, we compared this 3-year period with the most recent 3-year period available from the NCDB (2017-2019). We hypothesized that Hispanic and non-Hispanic Native American or Alaska Native individuals will have lower capture rates in the NCDB compared with the USCS database but that this capture rate will have improved over time.

## Methods

### Population

This study followed the Strengthening the Reporting of Observational Studies in Epidemiology (STROBE) reporting guideline for cohort studies (eFigures 1-2 in Supplement 1). This study was a retrospective analysis of all individuals who were diagnosed with prostate, breast, lung (including small cell, non–small cell, and other types), and/or colorectal cancer (including cancer at the colon, rectum, and rectosigmoid junction) between January 1, 2004, to December 31, 2006, and January 1, 2017, to December 31, 2019. We used the NCDB and USCS database. Exclusion criteria included individuals younger than 18 years. We also excluded individuals with missing race or ethnicity, with missing year of diagnosis, or who were diagnosed in other years. The University of California, San Diego determined this study was exempt from institutional review board approval and the informed consent process because all data were deidentified and publicly available.

### Data Collection

Data were collected using the NCDB 2020 Participant User File, which contains cases submitted to the CoC’s NCDB. The NCDB is a joint program as previously described between the ACS and American College of Surgeons. We secondarily collected data from the USCS database. The USCS database includes cancer statistics from the National Cancer Institute’s Surveillance, Epidemiology, and End Results Program and combines it with the Centers for Disease Control and Prevention’s National Program of Cancer Registries.^16^ It is estimated that the USCS database provides 100% of all cancer incidents in the US population.^16^

Data were subsequently acquired from the NCDB and USCS database and characterized according to year of diagnosis, cancer type, race, and ethnicity. Race and ethnicity were captured as self-reported information. For racial designation, individuals were grouped as American Indian or Alaska Native, Asian or Pacific Islander, Black, White, other (self-identified; the NCDB does not specify which races are counted under the other category), or unknown. For ethnicity, individuals were grouped as Hispanic or non-Hispanic, which included multiracial Hispanic. Race and ethnicity were combined to group individuals into the following self-identified categories: American Indian or Alaska Native, Asian or Pacific Islander, Black, Hispanic, White, and other or unknown. Capture rate was defined as the ratio of NCDB cases to USCS cases, and absolute percentage change (APC) was defined as the change in capture rates from 2004-2006 to 2017-2019. The primary outcome was the APC between the 2 comparison periods.

### Statistical Analysis

In this study, we used χ^2^ tests to determine whether the APC was significant. Statistical analyses were performed using SPSS software, version 28 (IBM Inc). A 2-sided *P* < .05 was considered to be statistically significant. Data analysis was performed from September 2022 to October 2023.

## Results

In total, 5 175 007 individuals (0.50% American Indian or Alaska Native, 3.10% Asian or Pacific Islander, 12.01% Black, 6.58% Hispanic, and 77.81% White) were diagnosed with breast, colorectal, lung, or prostate cancer in the USCS database between 2004-2006 and 2017-2019, and 3 242 964 of these individuals were also captured in the NCDB. When evaluating the overall capture rates for all individuals diagnosed with cancer in 2004-2006 and 2017-2019 (Table 1), the capture rate was lowest for individuals who were Hispanic (40.83% in 2004-2006 and 54.75% in 2017-2019; *P* < .001) and American Indian or Alaska Native (20.72% in 2004-2006 and 41.41% in 2017-2019; *P* < .001). Similarly, there were significant increases in the capture rates for both groups (Hispanic and American Indian or Alaska Native). There were also increases in the capture rate for Asian or Pacific Islander individuals, Black individuals, and White individuals. However, overall APC for Hispanic (13.92%) individuals remained less than the overall APC for White individuals (22.23%; *P* < .001).

**Table 1.** Changes in Overall Capture Rates of Breast, Colorectal, Lung, and Prostate Cancers Between 2004-2006 and 2017-2019 by Race and Ethnicity

Race and ethnicity	Capture rate, % (No./total No.)		Absolute percentage change, %	*P* value
	2004-2006	2017-2019		
American Indian or Alaska Native	20.72 (2149/10 368)	41.41 (6490/15 671)	20.69	<.001
Asian or Pacific Islander	50.46 (28 970/57 407)	71.39 (73 433/102 859)	20.93	<.001
Black	49.69 (136 978/275 689)	71.83 (248 329/345 741)	22.14	<.001
Hispanic	40.83 (53 855/131 889)	54.75 (114 317/208 785)	13.92	<.001
White	53.43 (1 051 709/1 968 415)	74.18 (1 526 734/2 058 183)	22.23	<.001
Mean	52.12 (1 273 661^a^^,^^b^/2 443 768^c^^,^^d^)	72.10 (1 969 303^e^^,^^f^/2 731 239^g^^,^^h^)	19.98	<.001

^a^ A total of 9477 individuals were removed because of unknown or other (self-identified; the National Cancer Database [NCDB] does not specify which races are counted under the other category) race.

^b^ A total of 11 5611 individuals were removed because of unknown ethnicity.

^c^ A total of 13 979 individuals were removed because of unknown or other (self-identified; the NCDB does not specify which races are counted under the other category) race.

^d^ A total of 5822 individuals were removed because of unknown or missing ethnicity.

^e^ A total of 18 245 individuals were removed because of unknown or other (self-identified; the NCDB does not specify which races are counted under the other category) race.

^f^ A total of 39 749 individuals were removed because of unknown ethnicity.

^g^ A total of 32 296 individuals were removed because of unknown or other (self-identified; the NCDB does not specify which races are counted under the other category) race.

^h^ A total of 6312 individuals were removed because of unknown or missing ethnicity.

In total, 1 702 627 individuals were diagnosed with breast cancer in the USCS database between 2004-2006 and 2017-2019, and 1 216 916 of these individuals were also captured in the NCDB (Table 2). When comparing capture rates for individuals diagnosed with breast cancer in 2004-2006 and 2017-2019, the capture rates were similarly lowest for individuals who were Hispanic or American Indian or Alaska Native. There was a significant increase in the capture rates for both groups (APCs of 13.28% for Hispanic and 21.61% for American Indian or Alaska Native; *P* < .001). There were also increases in the capture rate for Asian or Pacific Islander (APC, 18.35%), Black (APC, 20.82%), and White (APC, 17.90%) individuals (*P* < .001).

**Table 2.** Changes in Capture Rate of Breast Cancer Between 2004-2006 and 2017-2019 by Race and Ethnicity

Race and ethnicity	Capture rate, % (No./total No.)		Absolute percentage change, %	*P* value
	2004-2006	2017-2019		
American Indian or Alaska Native	25.17 (801/3182)	46.78 (2458/5254)	21.61	<.001
Asian or Pacific Islander	57.81 (12 851/22 229)	76.16 (36 246/47 588)	18.35	<.001
Black	60.23 (46 378/76 996)	81.05 (92 525/114 156)	20.82	<.001
Hispanic	47.20 (21 919/46 441)	60.48 (52 447/86 723)	13.28	<.001
White	63.44 (376 514/593 473)	81.35 (574 777/706 585)	17.90	<.001
Mean	61.76 (458 463^a^^,^^b^/742 321^c^^,^^d^	78.98 (758 453^e^^,^^f^/960 306^g^^,^^h^)	17.22	<.001

^a^ A total of 3107 individuals were removed because of unknown or other (self-identified; the National Cancer Database [NCDB] does not specify which races are counted under the other category) race.

^b^ A total of 47 324 individuals were removed because of unknown ethnicity.

^c^ A total of 3704 individuals were removed because of unknown or other (self-identified; the NCDB does not specify which races are counted under the other category) race.

^d^ A total of 1637 individuals were removed because of unknown or missing ethnicity.

^e^ A total of 7643 individuals were removed because of unknown or other (self-identified; the NCDB does not specify which races are counted under the other category) race.

^f^ A total of 15 781 individuals were removed because of unknown ethnicity.

^g^ A total of 7525 individuals were removed because of unknown or other (self-identified; the NCDB does not specify which races are counted under the other category) race.

^h^ A total of 2022 individuals were removed because of unknown or missing ethnicity.

In total, 878 797 individuals were diagnosed with colorectal cancer in the USCS database between 2004-2006 and 2017-2019, and 573 093 of these individuals were also captured in the NCDB (Table 3). When comparing capture rates for individuals diagnosed with colorectal cancer in 2004-2006 and 2017-2019, the capture rates were lowest for individuals who were American Indian or Alaska Native or Hispanic. Similarly, there were significantly increased capture rates for both groups (APCs of 11.78% for Hispanic and 19.10% for American Indian or Alaska Native; *P* < .001). There were also increases in the capture rate for Asian or Pacific Islander (APC, 19.62%), Black (APC, 18.46%), and White (APC, 17.77%) individuals (*P* < .001).

**Table 3.** Changes in Capture Rate of Colorectal Cancer Between 2004-2006 and 2017-2019 by Race and Ethnicity

Race and ethnicity	Capture rate, % (No./total No.)		Absolute percentage change, %	*P* value
	2004-2006	2017-2019		
American Indian or Alaska Native	22.15 (464/2095)	41.25 (1305/3164)	19.10	<.001
Asian or Pacific Islander	53.27 (6490/12 184)	72.89 (13 151/18 042)	19.62	<.001
Black	56.32 (28 187/50 047)	74.78 (39 383/52 664)	18.46	<.001
Hispanic	44.62 (12 197/27 337)	56.39 (23 530/41 724)	11.78	<.001
White	58.45 (208 648/356 990)	76.22 (239 738/314 550)	17.77	<.001
Mean	57.06 (255 986^a^^,^^b^/448 653^c^^,^^d^)	73.72 (317 107^e^^,^^f^/430 144^g^^,^^h^)	16.66	<.001

^a^ A total of 1705 individuals were removed because of unknown or other (self-identified; the National Cancer Database [NCDB] does not specify which races are counted under the other category) race.

^b^ A total of 26 395 individuals were removed because of unknown ethnicity.

^c^ A total of 1875 individuals were removed because of unknown or other (self-identified; the NCDB does not specify which races are counted under the other category) race.

^d^ A total of 1202 individuals were removed because of unknown or missing ethnicity.

^e^ A total of 3256 individuals were removed because of unknown or other (self-identified; the NCDB does not specify which races are counted under the other category) race.

^f^ A total of 6177 individuals were removed because of unknown ethnicity.

^g^ A total of 4044 individuals were removed because of unknown or other (self-identified; the NCDB does not specify which races are counted under the other category) race.

^h^ A total of 1021 individuals were removed because of unknown or missing ethnicity.

In total, 1 305 399 individuals were diagnosed with lung cancer in the USCS database between 2004-2006 and 2017-2019, and 751 400 of these individuals were also captured in the NCDB (Table 4). When comparing capture rates for individuals diagnosed with lung cancer in 2004-2006 and 2017-2019, the capture rates were lowest for individuals who were Hispanic or American Indian or Alaska Native. Similarly, there were significantly increased capture rates of both groups (APCs of 22.98% for Hispanic and 24.54% for American Indian or Alaska Native; *P* < .001). There were also increases in the capture rate for Asian or Pacific Islander (34.42%), Black (36.55%), and White (33.03%) individuals (*P* < .001).

**Table 4.** Changes in Capture Rate of Lung Cancer Between 2004-2006 and 2017-2019 by Race and Ethnicity

Race and ethnicity	Capture rate, % (No./total No.)		Absolute percentage change, %	*P* value
	2004-2006	2017-2019		
American Indian or Alaska Native	15.27 (416/2724)	39.81 (1697/4263)	24.54	<.001
Asian or Pacific Islander	36.53 (4210/11 524)	70.95 (14 601/20 580)	34.42	<.001
Black	38.28 (24 072/62 887)	74.83 (53 824/71 932)	36.55	<.001
Hispanic	30.53 (6666/21 831)	53.51 (17 052/31 866)	22.98	<.001
White	41.48 (218 657/527 198)	74.50 (410 205/550 594)	33.03	<.001
Mean	40.57 (254 021^a^^,^^b^/626 164^c^^,^^d^)	73.23 (497 379^e^^,^^f^/679 235^g^^,^^h^)	32.66	<.001

^a^ A total of 1320 individuals were removed because of unknown or other (self-identified; the National Cancer Database [NCDB] does not specify which races are counted under the other category) race.

^b^ A total of 28 819 individuals were removed because of unknown ethnicity.

^c^ A total of 1157 individuals were removed because of unknown or other (self-identified; the NCDB does not specify which races are counted under the other category) race.

^d^ A total of 1283 individuals were removed because of unknown or missing ethnicity.

^e^ A total of 3388 individuals were removed because of unknown or other (self-identified; the NCDB does not specify which races are counted under the other category) race.

^f^ A total of 9384 individuals were removed because of unknown ethnicity.

^g^ A total of 2300 individuals were removed because of unknown or other (self-identified; the NCDB does not specify which races are counted under the other category) race.

^h^ A total of 1569 individuals were removed because of unknown or missing ethnicity.

In total, 1 288 184 individuals were diagnosed with prostate cancer in the USCS database between 2004-2006 and 2017-2019, and 701 555 of these individuals were also captured in the NCDB (Table 5). When comparing capture rates for prostate cancer in 2004-2006 and 2017-2019, the capture rates were lowest for individuals who were American Indian or Alaska Native or Hispanic. However, the capture rate for Hispanic individuals significantly increased (APC, 7.88%), and a significant increase for American Indian or Alaska Native individuals (APC, 14.68%) was also noted (*P* < .001). Furthermore, there were similarly increased capture rates among the other racial and ethnic groups, including Asian or Pacific Islander (APC, 9.43%), Black (APC, 13.80%), and White (11.57%) individuals (*P* < .001).

**Table 5.** Changes in Capture Rate of Prostate Cancer Between 2004-2006 and 2017-2019 by Race and Ethnicity

Race and ethnicity	Capture rate, % (No./total No.)		Absolute percentage change, %	*P* value
	2004-2006	2017-2019		
American Indian or Alaska Native	19.77 (468/2367)	34.45 (1030/2990)	14.68	<.001
Asian or Pacific Islander	47.24 (5419/11 470)	56.67 (9435/16 649)	9.43	<.001
Black	44.71 (38 341/85 759)	58.51 (62 597/106 989)	13.80	<.001
Hispanic	36.03 (13 073/36 280)	43.92 (21 288/48 472)	7.88	<.001
White	50.51 (247 890/490 754)	62.08 (302 014/486 454)	11.57	<.001
Mean	48.70 (305 191^a^^,^^b^/626 630^c^^,^^d^)	59.91 (396 364^e^^,^^f^/661 554^g^^,^^h^)	11.21	<.001

^a^ A total of 3345 individuals were removed because of unknown or other (self-identified; the National Cancer Database [NCDB] does not specify which races are counted under the other category) race.

^b^ A total of 13 073 individuals were removed because of unknown ethnicity.

^c^ A total of 7243 individuals were removed because of unknown or other (self-identified; the NCDB does not specify which races are counted under the other category) race.

^d^ A total of 1700 individuals were removed because of unknown or missing ethnicity.

^e^ A total of 3958 individuals were removed because of unknown or other (self-identified; the NCDB does not specify which races are counted under the other category) race.

^f^ A total of 8407 individuals were removed because of unknown ethnicity.

^g^ A total of 18 427 individuals were removed because of unknown or other (self-identified; the NCDB does not specify which races are counted under the other category) race.

^h^ A total of 1700 individuals were removed because of unknown or missing ethnicity.

## Discussion

We present an analysis of the capture rates from the NCDB for individuals diagnosed with cancer based on their race and ethnicity for breast, colorectal, lung, and prostate cancer. For both time periods (2004-2006 and 2017-2019) across all 4 cancer types, the capture rates for Hispanic individuals and American Indian or Alaska Native individuals were significantly less than the capture rate for White individuals. These findings suggest that Hispanic and American Indian or Alaska Native individuals are significantly underrepresented in breast, colorectal, lung, and prostate cancer in the NCDB. However, substantial progress has been made in increasing representation since these disparities in the capture rates of Hispanic and American Indian or Alaska Native individuals were first reported more than 10 years ago. This progress is likely secondary to an increase in the number of CoC sites to more than 1500 facilities since its foundation in 1922. In addition, more individuals are seeking care at CoC sites.^3^

Across all cancer types in the NCDB, the APC for Hispanic individuals was consistently less than the APC for White individuals. Hispanic individuals experience significant barriers to care secondary to low socioeconomic status, language barriers, and immigration status.^10^ For Hispanic individuals diagnosed with cancer, additional factors, such as underrepresentation in clinical trials and transportation barriers, can create even more obstacles for detection and treatment of cancer.^17^ Furthermore, compared with non-Hispanic White individuals, Hispanic individuals have decreased breast cancer screening rates, higher levels of stress due to discrimination and costs of care, and decreased rates of health insurance coverage.^13,14^ The culmination of these factors leads to adverse health outcomes.^15^ Hispanic individuals are more likely non-Hispanic White individuals to live in medically underserved areas and, especially if part of a rural community, seek care at non–CoC-accredited facilities.^11,12^ Thus, these factors likely lead to a capture rate disparity.

In comparison, the APCs for American Indian or Alaska Native individuals were greater than the APCs for White individuals with prostate and breast cancers and less than the mean APC for lung cancer. American Indian or Alaska Native individuals are eligible for free care from the Indian Health Service (IHS), which was established in 1955 by the US federal government.^18^ However, the IHS has limited specialty services, funding, and sites, making it difficult for American Indian or Alaska Native individuals to access cancer care.^18,19^ Furthermore, 60% of American Indian or Alaska Native individuals live in urban areas that are far away from IHS centers, and many of them rely on private insurance to pay for health care or become uninsured; approximately 27% of all American Indian or Alaska Native individuals are uninsured.^19^ American Indian or Alaska Native individuals experience a mortality rate that is 18% greater than that of White individuals, despite a difference in overall cancer incidence rates of only 2%.^20^ This higher mortality may be partially attributed to underuse of cancer screening services, cultural barriers and medical mistrust, and lower likelihood of receiving care in accordance with guidelines.^19^ Expanding representation of American Indian or Alaska Native individuals in the NCDB is essential to addressing these barriers and improving outcomes for these individuals.

Structural racism has been identified as one of many variables associated with inequity in health care, and efforts to mitigate this barrier and increase access to care have been supported by many sources.^9^ The associations of Hispanic ethnicity and American Indian or Alaska Native race with NCDB capture rates have remained underexplored. Limited literature indicates that American Indian or Alaska Native individuals have the lowest capture rates across all genitourinary cancers.^7^ This finding is consistent with our findings. However, the literature does not explore these patterns for other cancer types or for Hispanic individuals and has not been updated since 2006.^7,8^ Nevertheless, the NCDB is widely used for many types of cancer research; in 2017, more than 300 articles were identified that used data from the NCDB.^21^ Many of these studies characterize the effects of clinical and socioeconomic factors on patient survival; thus, it is important that authors take cohort representation into consideration.^21^ An understanding of the limitations of the NCDB, including limited representation of Hispanic and American Indian or Alaska Native individuals, can help inform clinical decision-making, especially when using studies that are based on the NCDB. Such studies may underestimate disparities in care for these populations, and their findings may require extra consideration before being applied to Hispanic or American Indian or Alaska Native individuals.

### Limitations

Our study has several limitations. First, there were some individuals who had unknown race or ethnicity in the NCDB and USCS database; these individuals were excluded from this study. Furthermore, the USCS database does not provide information regarding the number of individuals who were of other races, and it does not specify which races are counted under the other category. Second, some individuals may have missing data in their medical records and were not captured by the NCDB.^22^ Third, the data were analyzed using coding, and errors in this process at contributing institutions collecting the data could lead to improper identification of patterns in the data. Fourth, the USCS database does not provide the stage of diagnosis or demographic baseline information (eg, sex distribution and mean age) for individuals diagnosed with cancer; therefore, analyses stratifying by stage of diagnosis or other demographic variables could not be performed in this study. In the end, this study presents the most updated information regarding NCDB capture rates for various races for breast, colorectal, lung, and prostate cancer.^7,8^

## Conclusions

In this cohort study of individuals diagnosed with cancer in the NCDB, capture rates in the NCDB were lowest for individuals who were Hispanic or American Indian or Alaska Native. However, there were significant improvements in representation of these individuals in the NCDB over time. These findings indicate that Hispanic and American Indian or Alaska Native representation has increased over time, but further efforts are needed.

## Notes

Supplement 1. **eFigure 1.** CONSORT Diagram for Individuals From NCDB Diagnosed With Breast, Colorectal, Lung, and Prostate Cancer **eFigure 2.** CONSORT Diagram for Individuals From USCS Database Diagnosed With Breast, Colorectal, Lung, and Prostate Cancer

Supplement 2.
Data Sharing Statement
