# Supplementary material for: Comparison of Capture Rates of the National Cancer Database Across Race and Ethnicity
Source: JAMA Netw Open. 2023 Dec 27;6(12):e2350237. doi: 10.1001/jamanetworkopen.2023.50237 (PMC10753391; doi:10.1001/jamanetworkopen.2023.50237)
Supplement: Supplement 1. — eFigure 1. CONSORT Diagram for Individuals From NCDB Diagnosed With Breast, Colorectal, Lung, and Prostate Cancer eFigure 2. CONSORT Diagram for Individuals From USCS Database Diagnosed With Breast, Colorectal, Lung, and Prostate Cancer [file jamanetwopen-e2350237-s001.pdf]

Supplementary Online Content

Satpathy Y, Nam P, Moldovan M, et al. Comparison of capture rates of the National Cancer Database across race and ethnicity. *JAMA Netw Open*. 2023;6(12):e2350237. doi:10.1001/jamanetworkopen.2023.50237

eFigure 1. CONSORT Diagram for Individuals From NCDB Diagnosed With Breast, Colorectal, Lung, and Prostate Cancer

eFigure 2. CONSORT Diagram for Individuals From USCS Database Diagnosed With Breast, Colorectal, Lung, and Prostate Cancer

This supplementary material has been provided by the authors to give readers additional information about their work.

© 2023 Satpathy Y et al. *JAMA Network Open.*

eFigure 1. CONSORT diagram for individuals from NCDB diagnosed with breast, colorectal, lung, and prostate cancer.

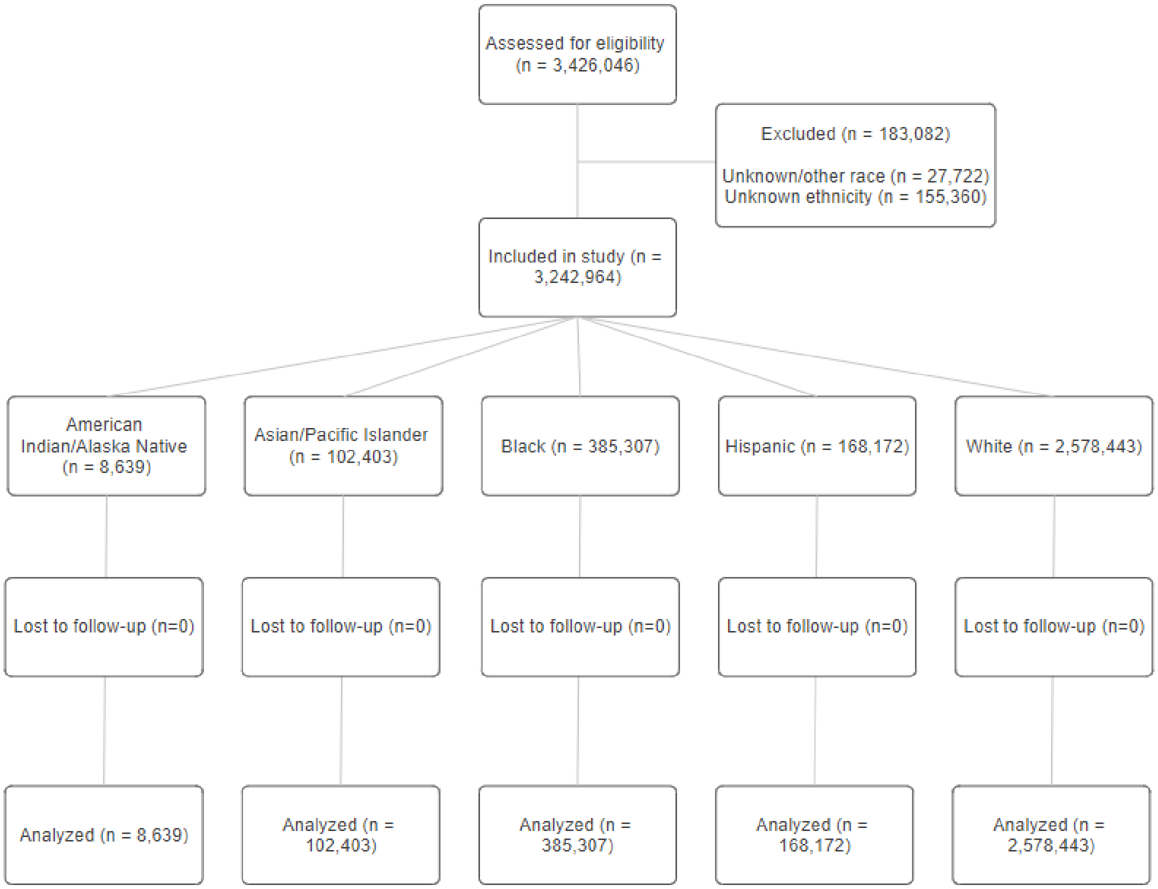

© 2023 Satpathy Y et al. *JAMA Network Open.*

eFigure 2. CONSORT diagram for individuals from USCS database diagnosed with breast, colorectal, lung, and prostate cancer.

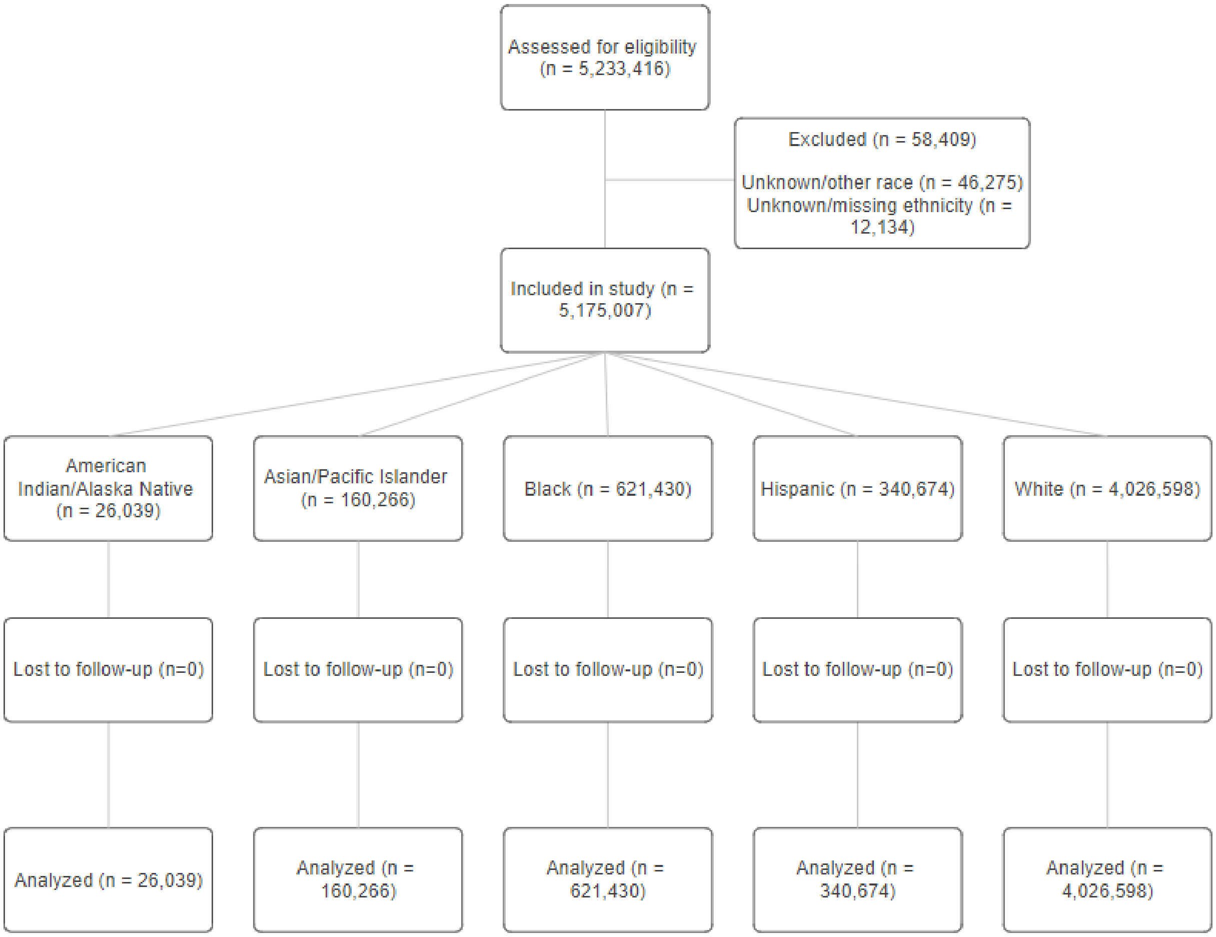

© 2023 Satpathy Y et al. *JAMA Network Open.*
