# Supplementary material for: Comparison of Capture Rates of the National Cancer Database Across Race and Ethnicity
Source: JAMA Netw Open. 2023 Dec 27;6(12):e2350237. doi: 10.1001/jamanetworkopen.2023.50237 (PMC10753391; doi:10.1001/jamanetworkopen.2023.50237)
Supplement: Supplement 2. — Data Sharing Statement [file jamanetwopen-e2350237-s002.pdf]

Data Sharing Statement

Satpathy. Comparison of Capture Rates of the National Cancer Database Across Race and Ethnicity. *JAMA Netw Open*. Published December 27, 2023.
doi:10.1001/jamanetworkopen.2023.50237

Data
Data available: No
